# Mechanical and Permeability Characteristics of Latex-Modified Pre-Packed Pavement Repair Concrete as a Function of the Rapid-Set Binder Content

**DOI:** 10.3390/ma8105339

**Published:** 2015-10-01

**Authors:** Jae-Woong Han, Ji-Hong Jeon, Chan-Gi Park

**Affiliations:** 1 Department of Bio-Industry Engineering, Koungju National University, Yesan 340-702, Korea; hanwoong@kongju.ac.kr; 2 Department of Environmental Engineering, Andong National University, 1375 Gyeongdong Street, Andong 760-749, Korea; jhjeon@anu.ac.kr; 3 Department of Rural Construction Engineering, Koungju National University, Yesan 340-702, Korea

**Keywords:** durability, mechanical properties, latex polymer, pavement repair concrete, rapid-set cement binder

## Abstract

We evaluated the strength and durability characteristics of latex-polymer-modified, pre-packed pavement repair concrete (LMPPRC) with a rapid-set binder. The rapid-set binder was a mixture of rapid-set cement and silica sand, where the fluidity was controlled using a latex polymer. The resulting mix exhibited a compressive strength of ≥21 MPa and a flexural strength of ≥3.5 MPa after 4 h of curing (*i.e.*, the traffic opening term for emergency repairs of pavement). The ratio of latex polymer to rapid-set binder material was varied through 0.40, 0.33, 0.29, and 0.25. Mechanical characterization revealed that the mechanical performance, permeability, and impact resistance increased as the ratio of latex polymer to rapid-set binder decreased. The mixture exhibited a compressive strength of ≥21 MPa after 4 h when the ratio of latex polymer to rapid-set binder material was ≤0.29. The mixture exhibited a flexural strength of ≥3.5 MPa after 4 h when the ratio of latex polymer to rapid-set binder material was ≤0.33. The permeability resistance to chloride ions satisfied 2000 C after 7 days of curing for all ratios. The ratio of latex polymer to rapid-set binder material that satisfied all conditions for emergency pavement repair was ≤0.29.

## 1. Introduction

There is growing demand for repairs to paving concrete and to minimize disruption to traffic the road should be re-opened as soon as possible [1,2,3,4,5]. There has therefore been much interest in design standards for materials used in emergency concrete repairs [6,7,8,9,10], and rapid-set cement concrete is the most commonly used material for this purpose [1,2,3,4,5]. When rapid-set cement is used, the large hydration heat increases the rate of vapor migration and may lead to high temperatures within the concrete itself. In practice, vapor migration may be restricted, which can lead to large tensile stresses and the formation of minute cracks [5,7,8,9,10]. These cracks increase the permeability of the pavement, leading to various forms of damage [7,8,9,10]. For these reasons, the structural integrity of the pavement may be compromised, requiring further repair [5,6,7,8,9,10]. The addition of a latex polymer can increase the workability of unhardened concrete and reduce the required ratio of water to cement [7,10]. This can also prevent separation of materials due to the viscosity of the latex polymer itself, which increases the flexural strength, tensile strength, and deformation capacity following hardening. This in turn improves the adhesion, water-tightness, resistance to freezing–thaw erosion, and improves the abrasion resistance and chemical resistance of the material [7,8,9,10].

Existing rapid-set cement latex-modified concretes are typically cast by mixing coarse aggregate, sand, cement, and water. Therefore, late-modified concrete is difficult to apply without a mobile mixer truck.

Here, this study used silica sand (*i.e.*, river sand) to replace the fine aggregates. A bonding material was applied to the pre-mixed rapid-set cement and silica sand. Pre-packed concrete is a construction method whereby a bonding material is injected into the coarse aggregates. Pre-packed concrete can be applied with a rapid set binder without requiring a mobile mixer.

This study investigated the performance of latex-polymer-modified, pre-packed pavement repair concrete (LMPPRC) incorporating a rapid-set binder. The LMPPRC construction method was as follows. The pavement to be repaired was removed and coarse aggregates were applied. The rapid-set binder material was mixed with a latex polymer and the resulting mix was poured onto the concrete between the coarse aggregates using a vibration compaction method. The mixture of latex polymer and rapid-set binder was adjusted to have sufficient fluidity to exhibit self-leveling behavior. Following infusion, the surface was finished to complete the repair. By mixing the latex polymer and the rapid-set binder in this manner, concrete repairs using LMPPRC can provide the required strength in a short time, with sufficient workability and durability. We evaluated the strength, permeability, and impact resistance of several LMPPRC formulations.

## 2. Materials

### 2.1. Materials

The rapid-set binder material used in this work was a mix of rapid-set cement and silica sands manufactured by Jung-Ang Polytec in Yangsan, Korea. The mixing ratio of rapid-set cement to silica sands was 1:1. Table 1 lists the chemical composition of the cement. Number 6 silica sand density, with a density of 2.65 g/mm^3^, was used. The crushed coarse aggregates had a maximum size of 25 mm and a density of 2.62 g/mm^3^. Table 2 lists the physical properties of the coarse aggregates. Table 3 lists the properties of the styrene butadiene (SB) latex polymer (Jung-Ang Polytec, Yangsan, Korea).

**Table 1:** Chemical compositions of the rapid set cement (Jung-Ang Polytec, Yangsan, Korea).

SiO_2_ (%)	Al_2_O_3_ (%)	Fe_2_O_3_ (%)	CaO (%)	MgO (%)	K_2_O (%)	SO_3_ (%)
13 ± 3	17.5 ± 3	>3	50 ± 3	>2.5	0.21	14 ± 3

**Table 2:** Physical properties of the coarse aggregates.

Properties	Density (g/mm^3^)	Absorption (%)	Fineness Modulus
Value	2.6	0.35	6.92

**Table 3:** Properties of the styrene butadiene latex.

Solids Content (%)	Styrene Content (%)	Butadiene Content (%)	Surface Tension (dyne/cm)	Particle Size (A)	Viscosity (cps)
8	34 ± 1.5	66 ± 1.5	30.57	1700	42

### 2.2. Mix Proportions

With concrete pavement repairs using the rapid-set cement, both the American Association of State Highway and Transportation Officials (AASHTO) and the Korean Highway Corporation require a maximum curing period of 4 h prior to re-opening the road to traffic [11,12]. The standards for traffic re-opening are a compressive strength of ≥21 MPa and a flexural strength of ≥3.5 MPa. Furthermore, after 28 days of curing, the compressive strength should be ≥35 MPa, the flexural strength should be ≥4.5 MPa, and the splitting tensile strength should be ≥4.2 MPa. Here we focused on concrete repairs using rapid-set cement, with the goal of achieving a compressive strength of ≥21 MPa and a flexural strength of ≥3.5 MPa after 4 h of curing, and a compressive strength of ≥35 MPa, flexural strength of ≥4.5 MPa, and a splitting tensile strength of ≥4.2 MPa after 28 days of curing. The permeability has a significant impact on the lifespan and durability of concrete pavement and we aimed for a permeability of ≤2000 C after 7 days of curing, which is recommended by the Korean Highway Corporation [12]. Chloride ion permeation tests were performed according to ASTM C1202 [13]. Table 4 lists the mixing ratios used in this work. 

### 2.3. Preparation of Test Specimens

Cylindrical specimens were formed by packing coarse aggregates into a cylindrical mold. The rapid-set cement and silica sands were mixed together, after which the latex polymer was added and mixed for 1 min and 30 s. Following mixing, the cylindrical specimen was placed on a vibration table and the rapid set binder materials were poured into the mix while the table was vibrating. Rectangular specimens were produced by packing the coarse aggregates into a rectangular frame. The rapid-set cement and silica sands were mixed together and the latex polymer was poured into the rapid-set binder material and mixed for one minute and 30 s. Following mixing, the rectangular specimens packed with coarse aggregates were placed on a vibration table, and the rapid set binder materials were poured into it while the table was vibrating.

**Table 4:** Mix proportions of the latex-polymer-modified, pre-packed pavement repair concrete (LMPPRC).

Type of Mix	Rapid Set Binder Materials (kg/m^3^)		Latex Polymer (kg/m^3^)	Coarse Aggregate (kg/m^3^)
	Rapid Set Cement	Silica Sand		
0.40	287.5	287.5	218	1450
0.33	313.5	313.5	210	1450
0.29	345	345	198	1450
0.25	366	366	184	1450

## 3. Test Methods

### 3.1. Compressive Strength Tests

Compressive strength tests were carried out in accordance with ASTM C 39 [14]. Tests were performed after curing periods of 4 h, 7 days, and 28 days. The specimens (100 mm × 200 mm) were cured in water at 23 ± 2 °C. Each variable was investigated using six specimens.

### 3.2. Flexural Tests

Flexural tests were carried out in accordance with ASTM C 78 [15]. Tests were carried out after curing periods of 4 h and 28 days. Specimens (100 mm × 100 mm × 400 mm) were cured in water at 23 ± 2 °C. Each variable was investigated using six specimens.

### 3.3. Splitting Tensile Tests

Splitting tensile tests were conducted in accordance with the ASTM C 496 standard [16]. Tests were carried out after curing periods of 4 h, 7 days, and 28 days. Specimens (100 mm × 200 mm) were cured in water at 23 ± 2 °C. Each variable was investigated using six specimens.

### 3.4. Chloride Ion Penetration Tests

**Figure 1:**
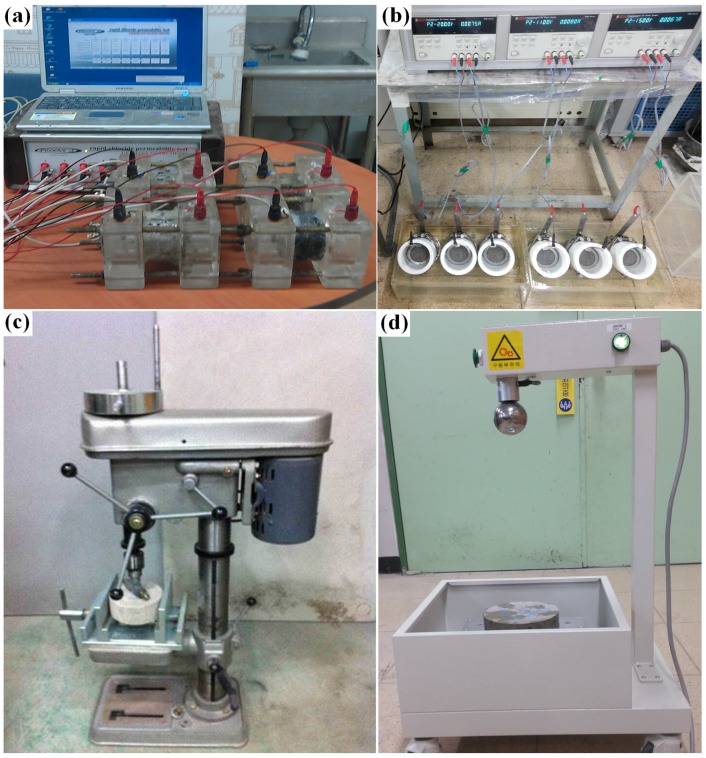
(**a**) Chloride ion penetration test set-up; (**b**) chloride ion diffusion test set-up; (**c**) abrasion test set-up; (**d**) impact test set-up.

Chloride ion penetration tests were conducted in accordance with ASTM C 1202 [13]. Specimens (150 mm × 50 mm) were cured in water at 23 ± 2 °C. Tests were carried out after curing periods of 4 h, 7 days, and 28 days. Each variable was investigated using six specimens. The test apparatus for the chloride ion penetration tests is shown in Figure 1a.

### 3.5. Chloride Ion Diffusion Tests

To investigate the diffusion characteristics of chloride ions, diffusion tests using fast electrophoresis were carried out after 28 days of curing using the NT Build 492 test standard of Northern Europe (NT Build 492, 1999) [17]. Specimens (100 mm × 50 mm) were cured in water at 23 ± 2 °C. Each variable was investigated using six specimens. Figure 1b shows the apparatus used to measure the chloride ion diffusion coefficient.

### 3.6. Abrasion Tests

Abrasion tests were carried out in accordance with ASTM C 944 [18]. Specimens (150 mm × 60 mm) were tested after curing for 28 days in water at 23 ± 2 °C. Each variable was investigated using six specimens. The test apparatus for the abrasion test is shown in Figure 1c.

### 3.7. Impact Tests

Impact testing was carried out in accordance with the specifications of ACI Committee 544 [19]. Specimens (150 mm × 60 mm) were cured in water at 23 ± 2 °C. Tests were carried out after a curing period of 28 days. Each variable was investigated using six specimens. Figure 1d shows the test set-up.

## 4. Results and Discussion

### 4.1. Compressive Strength

**Figure 2:**
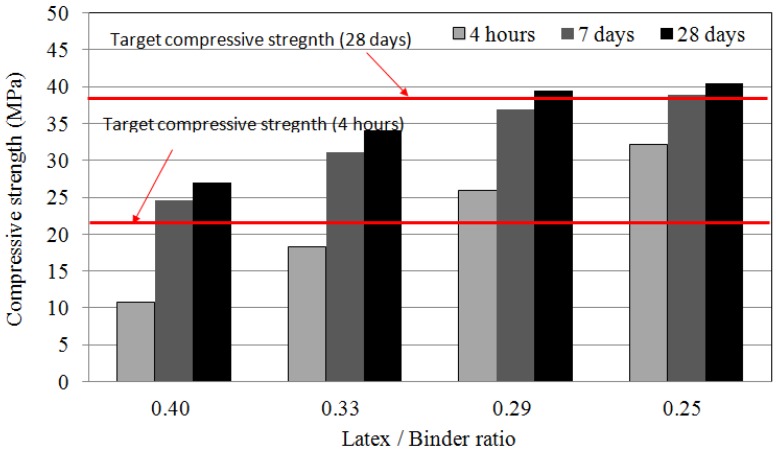
Compressive strength of latex-polymer-modified, pre-packed pavement repair concrete (LMPPRC).

The compressive strength of LMPPRC for emergency concrete repairs is shown in Figure 2. As the mixing ratio of latex polymer and rapid-set binder decreased, the compressive strength increased. When the ratio of latex polymer and rapid-set binder material was ≤0.29, the compressive strength satisfied the standard of 21 MPa after 4 h. The required compressive strength of 35 MPa was satisfied after curing for 28 days with a mixing ratio of ≤0.29. It appears that the presence of the latex polymer retarded the manifestation of early strength, leading to a failure to satisfy the standard. Furthermore, when the quantity of rapid-set binder was small, sufficient early strength could not be obtained. Therefore, to satisfy both goals of retarding the appearance of strength using latex polymer and to acquire sufficient early strength, it was necessary to increase the quantity of rapid-set binder. When the ratio of latex polymer to rapid-set binder was ≤0.29, the mix was able to satisfy the standards for emergency concrete repair.

### 4.2. Flexural Strength

**Figure 3:**
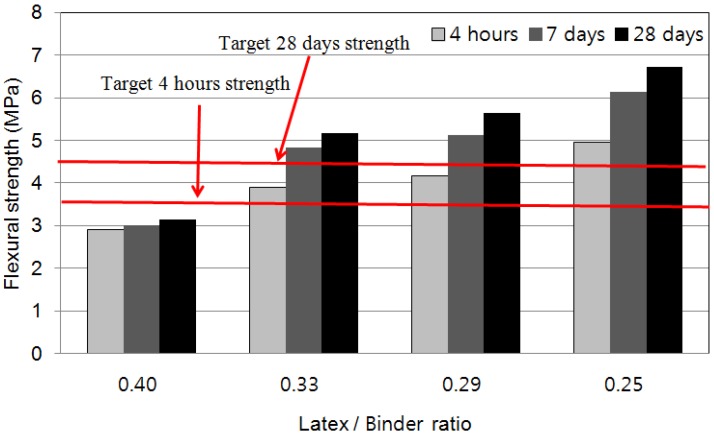
Flexural strength of LMPPRC.

Figure 3 shows the results of flexural strength tests for the LMPPRC for emergency repairs. The standard for flexural strength was 3.5 MPa after 4 h (for traffic re-opening) and 4.5 MPa after 28 days. When the mixing ratio of latex polymer to rapid-set binder material was ≤0.33, the concrete satisfied 3.5 MPa after 4 h and 4.5 MPa after 28 days. This shows that the addition of latex polymer to concrete had more influence on the tensile strength and flexural strength than on the compressive strength. The presence of the latex polymer increases the binding force between materials when the concrete is subjected to flexural or tensile stresses [7,8,10] Therefore (unlike the compressive strength), the flexural strength was sufficiently large to satisfy the standards for emergency concrete repair even when the ratio of latex polymer to rapid-set binder material was 0.33. Furthermore, the flexural strength increased as the ratio of latex polymer to rapid-set binder decreased.

### 4.3. Splitting Tensile Strength

**Figure 4:**
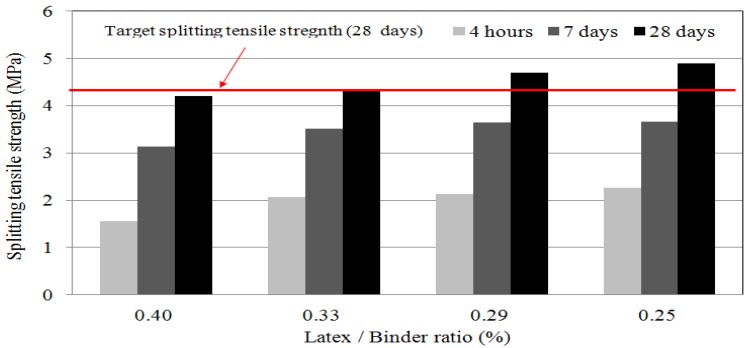
Splitting tensile strength of LMPPRC.

The standard for splitting tensile strength of LMPPRC was 4.2 MPa after 28 curing for days, as defined by the Korean Highway Corporation. Splitting tensile strength increased as the ratio of latex polymer to rapid-set binder decreased (see Figure 4). A tensile strength of 4.2 MPa after curing for 28 days was satisfied when the mixing ratio was ≤0.29, but not with mixing ratios of 0.40 and 0.33. This shows that the addition of the latex polymer to concrete had more influence on the tensile strength than on the compressive strength. The latex polymer effectively increased the binding force between the materials when the concrete was subjected to tensile stress, which enhanced the tensile strength. In general, the tensile strength of concrete is lower than its flexural strength, which is consistent with the findings of this work.

### 4.4. Chloride Ion Penetration

**Figure 5:**
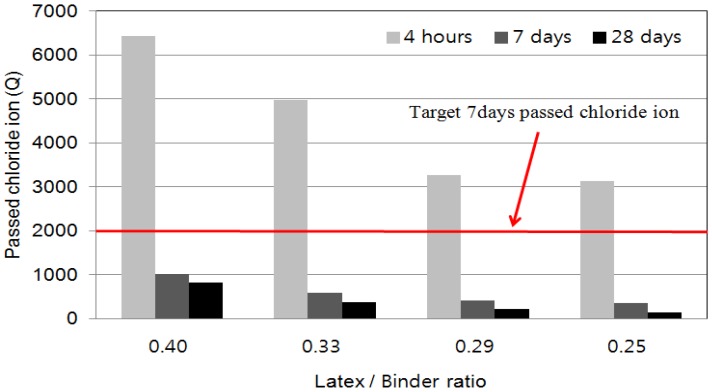
Chloride ion penetration test results of LMPPRC.

Figure 5 shows the chloride ion permeability resistance of LMPPRC. The permeability standard was ASTM C 1202 (see Table 5) [17]. Chloride ion permeability decreased as the ratio of latex polymer to rapid-set binder decreased. Comparing these data with the ASTM C 1202 standard, we can see that the chloride ion permeability after 4 h was “high” for latex polymer/rapid-set binder ratios of 0.40 and 0.33 (*i.e.*, ≥4000 C). With latex polymer/rapid-set binder ratios of 0.29 and 0.25, the chloride ion permeability was “moderate” (*i.e.*, 2000–4000 C). After 7 days of curing, the chloride ion permeability was “low” for a latex polymer/rapid-set binder ratio of 0.40 (*i.e.*, 1000–2000 C), whereas for latex polymer/rapid-set binder ratios of 0.33, 0.29, and 0.25, the permeability was “very low” (*i.e.*, ≤1000 C). After 28 days of curing, all the specimens exhibited a “very low” chloride permeability (*i.e.*, 100–1000 C). After 7 days of curing, the target chloride ion permeability resistance of 2000 C was achieved with all the ratios of latex polymer/rapid-set binder.

The addition of latex polymer reduced the permeability of chloride ions by filling pores and forming a thick latex polymer network in the concrete. Therefore, all the specimens exhibited excellent permeability resistance. Furthermore, an increase in strength generally reduces chloride ion permeability [7,8,10]. However, after curing for 4 h, the strength was insufficient. Moreover, internal cracking was initiated during the preparation of samples for chloride ion penetration tests. These cracks propagated, leading to an increase in the permeability of the LMPPRC.

**Table 5:** The ASTM C 1202 chloride ion permeability classifications.

Charge Passed (C)	Chloride Permeability
>4000	High
2000–4000	Moderate
1000–2000	Low
100–1000	Very low
<100	Negligible

### 4.5. Chloride Ion Diffusion

**Figure 6:**
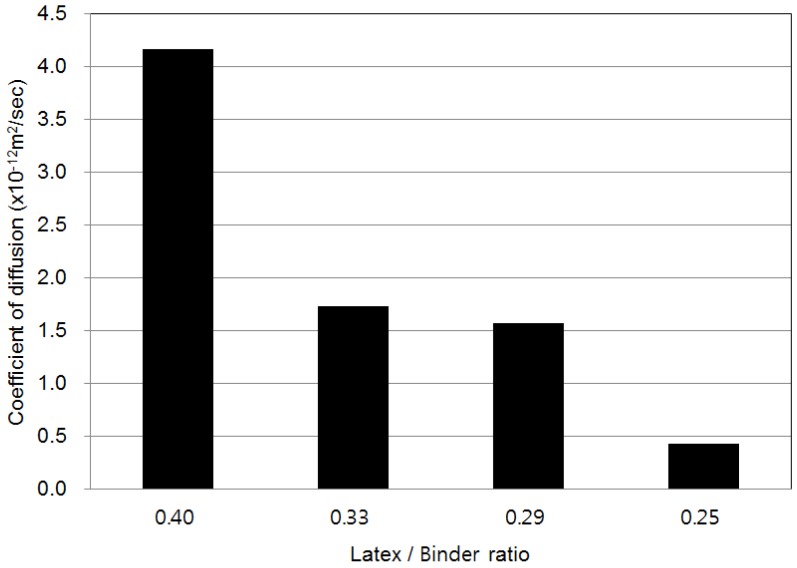
Chloride ion diffusion test results of LMPPRC.

Chloride ion diffusion coefficients were obtained from the results of fast electrophoresis tests using the model of Tang and Nilsson (1992) [20]. Figure 6 shows the chloride ion diffusion coefficient of various LMPPRCs. The diffusion coefficient decreased as the ratio of latex polymer to rapid-set binder decreased. The chloride ion diffusion tests exhibited results similar to those of the chloride ion permeability tests. As the quantity of rapid set binder increased, the strength of the LMPPRC increased, which was attributed to a reduction in the number of internal pores. Furthermore, the increase in strength reduced permeability through improvements in the microstructure of the concrete, which appear to have led to decreases in the chloride ion diffusion coefficients.

### 4.6. Abrasion Resistance

**Figure 7:**
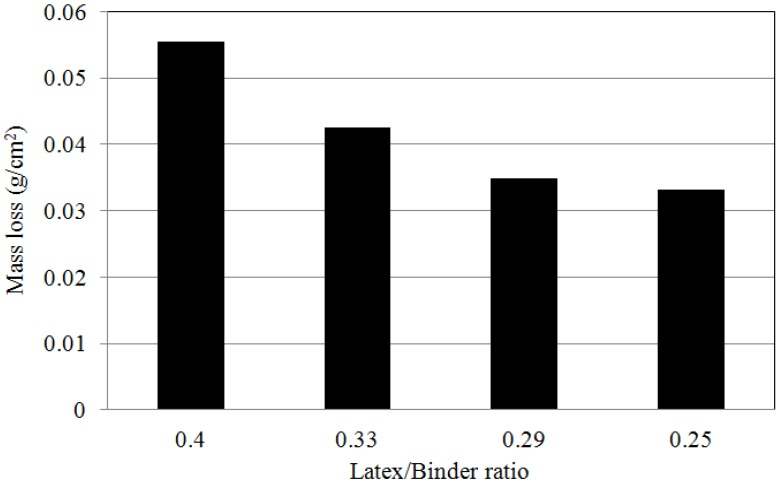
The results of abrasion resistance tests for LMPPRC.

Figure 7 shows the abrasion resistance of the LMPPRCs with varying quantities of rapid set binder for use in concrete pavement repair. Abrasion resistance increased as the quantity of rapid set binder increased. This was attributed to an increase in the strength of the surface. Additionally, the bond between materials was improved as the quantity of rapid-set binder increased. Furthermore, in increase in the quantity of binder resulted in an increase in the stiffness of the concrete, which increased the resistance to abrasion.

### 4.7. Impact Resistance

**Figure 8:**
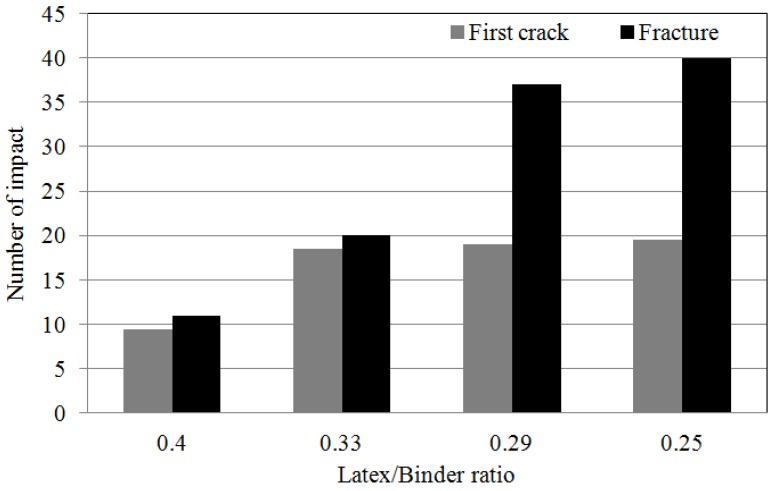
The results of impact tests for the LMPPRC.

Figure 8 shows the measured impact resistance of the LMPPRC for applications in the repair of concrete pavement. The number of impacts prior to the first occurrence of cracking increased as the quantity of rapid set binder increased, as did the number of impacts prior to failure. This is because the rapid set binder improved both the strength of the LMPPRC and the integrity of the bonding interface between materials, which inhibited crack formation and propagation.

## 5. Conclusions

In this study, we evaluated the strength and permeability characteristics of LMPPRCs with various ratios of latex polymer to rapid-set binder. The main results of this work can be summarized as follows.

When the ratio of latex polymer to rapid-set binder was ≤0.29, the LMPPRC exhibited a compressive strength of ≥21 MPa after curing for 4 h, and of ≥35 MPa after curing for 28 days. This satisfied the design standard for emergency repair concrete.

Flexural strength tests showed that the mixing ratio of latex polymer to rapid-set binder material should be ≤0.33 to provide a flexural strength of ≥3.5 MPa after 4 h and a flexural strength of ≥4.5 MPa after 28 days.

Splitting tensile strength tests showed that the ratio of latex polymer to rapid-set binder material should be ≤0.29 to provide a tensile strength of ≥4.2 MPa after 28 days.

Chloride ion permeability tests showed that all of the evaluated ratios of latex polymer to rapid-set binder satisfied the standard of 2000 C. As the ratio of latex polymer to rapid-set binder decreased, the permeability of chloride ions decreased.

These data show that diffusion coefficients decreased, impact resistance increased, and abrasion resistance increased as the ratio of latex polymer to rapid-set binder decreased. This was attributed to an increase in strength of the LMPPRC due to the presence of the rapid-set binder, which was in turn attributed to a decrease of the number of internal pores in the concrete. Increasing the quantity of rapid-set binder reduced the number of pores in the concrete, thereby reducing the chloride ion diffusion coefficient and increasing the impact resistance while reducing the rate of abrasion.
